# Untargeted plasma metabolomics and risk of colorectal cancer—an analysis nested within a large-scale prospective cohort

**DOI:** 10.1186/s40170-023-00319-x

**Published:** 2023-10-17

**Authors:** Linda Vidman, Rui Zheng, Stina Bodén, Anton Ribbenstedt, Marc J. Gunter, Richard Palmqvist, Sophia Harlid, Carl Brunius, Bethany Van Guelpen

**Affiliations:** 1 Department of Radiation Sciences, Oncology, Umeå University, Umeå, Sweden; 2 Department of Surgical Sciences, Medical Epidemiology, Uppsala University, Uppsala, Sweden; 3 Department of Clinical Sciences, Pediatrics, Umeå University, Umeå, Sweden; 4 Department of Biology and Biological Engineering, Chalmers University of Technology, Gothenburg, Sweden; 5 Chalmers Mass Spectrometry Infrastructure, Chalmers University of Technology, Gothenburg, Sweden; 6 Nutrition and Metabolism Branch, International Agency for Research on Cancer, World Health Organization, Lyon, France; 7 Department of Epidemiology and Biostatistics, School of Public Health, Imperial College London, London, UK; 8 Department of Medical Biosciences, Pathology, Umeå University, Umeå, Sweden; 9 Wallenberg Centre for Molecular Medicine, Umeå University, Umeå, Sweden

**Keywords:** Untargeted metabolomics, Colorectal cancer, Early detection

## Abstract

**Background:**

Colorectal cancer (CRC) is a leading cause of cancer-related death worldwide, but if discovered at an early stage, the survival rate is high. The aim of this study was to identify novel markers predictive of future CRC risk using untargeted metabolomics.

**Methods:**

This study included prospectively collected plasma samples from 902 CRC cases and 902 matched cancer-free control participants from the population-based Northern Sweden Health and Disease Study (NSHDS), which were obtained up to 26 years prior to CRC diagnosis. Using reverse-phase liquid chromatography–mass spectrometry (LC–MS), data comprising 5015 metabolic features were obtained. Conditional logistic regression was applied to identify potentially important metabolic features associated with CRC risk. In addition, we investigated if previously reported metabolite biomarkers of CRC risk could be validated in this study population.

**Results:**

In the univariable analysis, seven metabolic features were associated with CRC risk (using a false discovery rate cutoff of 0.25). Two of these could be annotated, one as pyroglutamic acid (odds ratio per one standard deviation increase = 0.79, 95% confidence interval, 0.70–0.89) and another as hydroxytigecycline (odds ratio per one standard deviation increase = 0.77, 95% confidence interval, 0.67–0.89). Associations with CRC risk were also found for six previously reported metabolic biomarkers of prevalent and/or incident CRC: sebacic acid (inverse association) and L-tryptophan, 3-hydroxybutyric acid, 9,12,13-TriHOME, valine, and 13-OxoODE (positive associations).

**Conclusions:**

These findings suggest that although the circulating metabolome may provide new etiological insights into the underlying causes of CRC development, its potential application for the identification of individuals at higher risk of developing CRC is limited.

**Supplementary Information:**

The online version contains supplementary material available at 10.1186/s40170-023-00319-x.

## Background

Colorectal cancer (CRC) is the second leading cause of cancer-related death worldwide [1]. CRC tends to progress slowly without clear symptoms at early stages, and the high number of deaths due to CRC is partly a consequence of late detection. Thus, effective screening methods for early detection and for removal of precancerous lesions are essential to decrease CRC mortality. Development of a blood-based screening method could play an important role in motivating individuals at risk to undergo further screening, thereby reducing CRC incidence and mortality.

Metabolomics is the study of small molecules (metabolites) that are present in biological systems at a given time point. The metabolomic profile can reflect extrinsic exposures, such as diet and tobacco, as well as intrinsic factors, including genetic variation [2]. Metabolic reprogramming is an important cancer hallmark, and metabolomics is currently being used both to discover diagnostic disease biomarkers and to investigate etiological pathways involved in cancer development, which might have clinical implications for targeted pharmaco-prevention or therapy [3].

Previous studies have identified differences in metabolomic profiles between cases and healthy controls for several cancer types including CRC [4, 5]. A Chinese study of 22 colon cancer patients, 23 rectal cancer patients, and 45 healthy control participants reported differences in the levels of several serum metabolites between the three groups, and comparisons between preoperative and postoperative samples indicated that changes in the metabolic profiles were associated with the outcome of surgical treatment [6]. However, few studies have identified biomarkers of etiology or for early detection of CRC based on metabolic profiles in early-stage CRC or in pre-diagnostic settings, such as samples collected at screening or in prospective cohorts [7]. A prospective study in an Asian population identified 35 metabolites associated with subsequent CRC risk [8], none of which was replicated in a study based on the Cancer Prevention Study II Nutrition Cohort [9]. Six metabolites were associated with CRC risk in the latter study, which remain to be validated.

In this study, we employed an untargeted metabolomics approach, using pre-diagnostic blood samples from 902 CRC cases and 902 individually matched control participants from a population-based cohort, to investigate the potential of plasma-based metabolomics for prediction of CRC risk. In addition, we investigated whether previously reported metabolite biomarkers of prevalent and/or incident CRC could be validated in our study.

## Methods

### Study population

The study population was derived from two population-based cohorts within the Northern Sweden Health and Disease study (NSHDS). The majority of the study participants (91%) were part of the Västerbotten Intervention Programme (VIP), which invites the residents in Västerbotten County to general health exams. The study intends to invite all residents at 10-year intervals at 40, 50, and 60 years of age (and 30 years of age until 1996), and the participation rates have varied over time with an average of around 60%. The physical exam includes measurements of height, weight, blood pressure, blood lipids, and an oral glucose tolerance test, and the participants are asked to donate a blood sample for biobanking. Participants in VIP complete extensive questionnaires regarding health and health-related factors, such as lifestyle. The remainder of the data (9%) were collected from the WHO’s Northern Sweden Multinational Monitoring of Trends and Determinants in Cardiovascular Disease (MONICA) study. Using a random selection of participants aged 25–74 years from the counties of Västerbotten and Norrbotten and repeated every 4–5 years since 1986, the MONICA study followed very similar protocols to the VIP [10]. Both cohorts have been described in detail elsewhere [11, 12]. Around 20% (374 participants) of the 1804 individuals in this study had a repeated measure, meaning that they participated in an NSHDS cohort on two occasions, for example, at 50 and 60 years of age.

### Sample collection and storage

The blood samples were taken after 5 min of rest and after more than 8 h of fasting for most subjects (83%). The blood samples used in this study were collected in EDTA tubes, and aliquots of plasma, buffy coat, and erythrocytes were frozen (− 20 $^\circ \mathrm{C}$) within 1 h of collection. Within a week, the tubes were transported to − 80 $\mathrm{^\circ{\rm C} }$ freezers at a central storage facility.

### Selection of study participants

Cohort participants who later developed CRC were identified through linkage with the Swedish Cancer Registry. Individuals that participated in MONICA or VIP before 19th January 2016 were subject for inclusion, and the cut-off date for case diagnosis was on 31st May 2016. Tumor stage and anatomical tumor location data were retrieved through the Swedish Colorectal Cancer Registry and, when necessary, through individual patient records. Tumor site was defined using the International Classification of Disease 10th edition (ICD-10) codes: C.18.0 and C 18.2–18.4 for proximal colon, C18.5–18.7 for distal colon, and C19.9 and C20.9 for rectum. Exclusions were made for participants with previous cancer diagnoses other than non-melanoma skin cancer. Individual matching of cases and controls was based on the following: sex, age at baseline (± 1 year), cohort, year of blood sampling and data collection (± 1 year), number of freeze–thaw cycles of the plasma samples (92% with exact match), and fasting status at blood collection. All samples, including the repeated measures were prediagnostic. For participants with repeated sampling occasions, the sample collected closest to the diagnosis date of the CRC case was used for each case set in the main analyses.

### Tumor tissue analysis

Formalin-fixed and paraffin-embedded CRC tissue samples were collected either through primary tumor resection (majority of the samples) or biopsies (small proportion of the samples) at Umeå University Hospital, Sweden. *KRAS* and *BRAF* mutational status was determined by analyzing DNA extracted using a Qiagen QIAamp DNA FFPE Tissue Kit. Sequencing to determine *KRAS* mutation (codon 12 and 13) was carried out using BigDye v. 3.1 (Applied Biosystems, Life Technologies, Stockholm, Sweden). *BRAF* mutations were detected by TaqMan allelic discrimination assay or digital droplet PCR. Tumors were determined as either microsatellite instable (MSI) or microsatellite stable (MSS) using immunohistochemical analysis or a PCR-based method. The tumor tissue analyses are described in more detail elsewhere [13].

### Metabolomic profiling

Plasma samples were aliquoted and sorted to preserve case sets, with random ordering within sets, and cold-shipped (− 80 °C) to the Chalmers Mass Spectrometry Infrastructure at Chalmers University of Technology, Gothenburg, Sweden. Samples were thawed at 4 °C, vortexed, and an aliquot of 30 µL together with 200 µL of cold acetonitrile (ACN, VWR International) was added to the well of a 96-deep well microplate (Captiva, Agilent Technologies), which was then mixed on an orbital shaker for 3 min at 1000 rpm. The microplate was centrifuged for 10 min at 500 g at 4 °C, and the supernatant was filtered through a 96-well filter plate (0.45 µm, Captiva, Agilent Technologies). The filtrate was collected in a 96-well microplate (Nunc, Thermo Fisher Scientific), which was centrifuged at 500 g at 4 °C for 1 min and kept at 4 °C until instrumental analysis. The preparation of study-specific quality control samples (sQCs) was conducted by pooling equal amounts of plasma from samples in the first two batches. The sQCs were subject to the same sample preparation procedure as the actual samples. sQCs were injected at the beginning, at the end, and systematically between every 11 samples throughout the batch sequence. Independent long-term quality control plasma samples (ltQCs) were used to monitor the performance of the instrument and to correct for batch effects [14].

The analysis of plasma samples was performed on an Agilent UHPLC-qTOF-MS system which consisted of a 1290 Infinity series UHPLC system with a 6550 UHD iFunnel accurate-mass qTOF spectrometer. During the analysis, the sample chamber was kept at 4 °C. Metabolites were separated by reversed-phase chromatography on a Waters ACQUITY UPLC HSS T3 column (100 × 2.1 mm, 1.8 µm). The Agilent MassHunter workstation was used to operate and monitor the instrument and acquire data. The mobile phase included (A) water and (B) methanol, both containing 0.04% formic acid. The linear gradient elution was as follows: 0–6 min, 5–100% B and 6–10.5 min, 100% B. The flow of mobile phase was set at 0.4 mL/min. Metabolites were ionized by a Jet Stream Electrospray ionization (ESI) source. The mass spectrometer was operated in both positive and negative modes, and 2 and 4 µL of sample were injected for positive and negative modes, respectively. The spectrometer parameters were set as follows: drying gas (nitrogen) temperature at 175 °C and flow at 12 mL/min, sheath gas temperature at 350 °C and flow at 11 L/min, nebulizer pressure at 45 psi, capillary voltage at 3500 V, nozzle voltage at 300 V, and fragmentor voltage at 175 V. Data were acquired within mass-to-charge ratio (m/z) 50–1600 in centroid mode with the acquisition rate at 1.67 spectra/s. The MS abundance threshold was set at 200. Iterative MS/MS data acquisition was performed on sQC samples in both modes with 10, 20, and 40 eV collision energies and with the same chromatographic conditions as for the MS analysis.

### Metabolomics data preprocessing

Vendor raw data files were converted into mzML format (Proteo Wizard, version 3.0) for data preprocessing, which was mainly performed using the R package “XCMS” [15]. Data from reversed-phase positive (RP) and negative (RN) modes were processed separately. The key parameters of XCMS were optimized with the aid of the R package “IPO” [16]. In total, 8236 metabolite features were obtained for RP and 6599 features for RN. Imputation for missing values in the metabolomics data was conducted using an in-house random forest-based algorithm (the mvImpWrap function from https://gitlab.com/CarlBrunius/StatTools). Within- and between-batch normalization were performed using the R package “BatchCorr” [14]. After normalization, 4804 features for RP and 4461 features for RN with coefficient of variation (CV) ≤ 30% among sQCs were retained. Finally, features presumably derived from the same metabolite were grouped with the R package “RAMClustR” [17] using manually optimized parameters. The feature with the highest intensity in each group was selected to represent the corresponding metabolite. Features not grouped were retained as singletons. The final data set comprised 2644 features for RP and 2391 features for RN. Parameters used for XCMS and RAMClustR are presented in additional notes.

### Metabolite identification

Metabolite identification was carried out using an in-house native standard library and the MassBank of North America [18], as well as the in *silico* fragmentation tools MetFrag [19] and SIRIUS [20]. All files containing MS2 spectra were converted to.mgf format prior to analysis. Identification was carried out according to the Schymanski scale, determining the confidence level (CL) on a scale from 1 to 5 [21]. We considered CL 1 (confirmed structure by reference match) for comparisons against in-house reference library with a modified cosine score above 0.9; CL 2 (probable structure by library/diagnostic evidence) for an exact spectral similarity above 0.9 in MetFrag; CL 3 (tentative candidate) for features where the majority of spectra of a feature were predicted to be the same compound by both MetFrag and SIRIUS; CL 3 or CL 4 (unequivocal molecular formula) was assigned, depending on manual assessment of spectral similarity when the majority of the spectra of a feature were predicted to be the same compound in either MetFrag or SIRIUS, but not by both; CL 4 was also assigned when the majority of the spectra were predicted to have the same chemical formula in SIRIUS; and CL 5 (mass of interest) was assigned when no MS2 was obtained for a feature or when there was no majority of spectral predictions. Parameters for SIRIUS, MetFrag, HMDB, and in-house library matching are found in additional notes.

### Statistical analysis

All statistical analyses were performed in R v4.0.3 [22]. A *p*-value below 0.05 was considered statistically significant. When testing differences in metabolite features by CRC status, the false discovery rate (FDR) was controlled for using the Benjamini–Hochberg method [23]. A relatively non-stringent FDR cutoff of 0.25 was chosen to increase the possibility to find potential biomarkers in the exploratory main analysis. Baseline characteristics of the study participants were presented as mean values and standard deviations or as frequencies and percentages. Differences between matched cases and controls were assessed using paired Wilcoxon signed-rank tests or chi-squared tests. Metabolite-wise analysis of case–control status was performed using conditional logistic regression to identify metabolite features associated with CRC risk. Odds ratios (OR) were calculated per 1 standard deviation increase in the metabolite feature and expressed with a 95% confidence interval (CI). Significant features were also studied in the earlier samples from the 374 individuals with repeated sample occasions.

Classification of participants according to disease status and clinical and molecular tumor subtypes was performed using a random forest model with incorporated recursive variable selection in a repeated double cross-validation procedure (R package MUVR) [24]. Parameters in the models were set to the following: *varRatio* = 0.85, *nOuter* = 6, and *nrep* = 30. Separate models were constructed based on time intervals from sampling to diagnosis according to < 5 years prior to diagnosis, 5–9 years prior to diagnosis, 10–15 years prior to diagnosis, and > 15 years prior to diagnosis. Sensitivity analysis was performed by stratifying by sex as well as limiting to participants who had fasted > 8 h. Subgroup analysis was conducted by considering CRC cases defined by the following: tumor location (proximal colon, distal colon, rectum), tumor stage (stages I–II, stages III–IV), *KRAS* mutation (mutation, wild type), *BRAF* mutation (mutation, wild type), combined *KRAS*/*BRAF* mutation status (*KRAS* mutated, *BRAF* mutated, both wild type), and MSI status (MSI, MSS). A set of a priori defined potential confounders with adequate data available was included together with the metabolomics data for selection in the random forest models. The aim in a random forest model is to reduce classification error by splitting the data so that the variance is minimized. The model does not consider the confounding effect of covariates in the split of the data, so the potential confounders were added to the model to see if they were retained and could improve predictions. The potential confounders included the following: body mass index (BMI, kg/m^2^), smoking status (current smoker, former smoker or nonsmoker), education level (elementary school (9 years of compulsory school for children aged 7 post-secondary 16), secondary school, postsecondary education), diabetes (yes or no, self-reported in questionnaire), alcohol intake (g/day trichotomized to the following: zero intake, intake below sex-specific median or above sex-specific median), and recreational physical activity (single questionnaire item: never, now and then, 1–2 times/week, 2–3 times/week, > 3 times/week).

Two random forest models were built for each subgroup analysis. One was based only on the metabolomics profiles, and a second model included the abovementioned potential confounders as well as the case–control matching variables (cohort, baseline age, sex, year of blood sampling, fasting status, and number of freeze and thaw cycles) together with the metabolite features.

The predictive power was assessed by calculating overall error rate (OER) and balanced error rate (BER) using predictions from cross validation, where $\mathrm{BER }=\frac{\sum_{Class}{Error Rate}_{Class}}{{n}_{Class}}$ and $\mathrm{OER}=1-\mathrm{correct classification rate}$. Consequently, BER under random conditions vary depending on the number of subgroups. For three subgroups, the expected BER_random_ is 0.67, and for two subgroups, the expected BER_random_ is 0.5. Expected OER under random conditions were obtained by randomly permuting the class labels (*n* = 10,000). Imputation of missing data among confounder variables was handled by replacing the missing value with the sex-specific mode for discrete variables and with the sex-specific median for continuous variables. Median values and modes were calculated based on the data in this study. For categorical variables in which more than 10% of the samples had missing values, the missing values were considered as a dummy category in the model.

### Replication of previously reported CRC biomarker candidates

Metabolites previously reported to be associated with incident and/or prevalent CRC were identified through a literature search in PubMed in March 2021 for studies on metabolite biomarkers for CRC (Additional Table 1). Identification of candidate masses in our metabolomics data was performed using exact mass when reported in the original publications. In addition, monoisotopic masses for all candidates were extracted from HMDB, and theoretical m/z-values were obtained for positive and negative ionization-specific adducts, respectively: [M + H]^+^, [M + Na]^+^, [M + K]^+^, [M + NH_4_]^+^, [M + 2H]^+^, [M + ACN + H]^+^, and [M − H_2_O + H]^+^ for positive ionization and [M − H]^−^, [M − H_2_O − H]^−^, [M − 2H]^−^, [M + Na − 2H]^−^, [M + K − 2H]^−^, [M + Cl]^−^, and [M + FA − H]^−^ for negative ionization. Metabolite candidates were considered to be replicated in our study for features that both corresponded to a lookup match (within class tolerance < 10 ppm) and were associated with CRC risk in multivariable conditional logistic regression adjusted for the potential confounders described above (nominal *p* < 0.05). The candidate list was filtered to remove instrument artifacts, isotopes, or features having an MS/MS fragmentation pattern not corresponding to the candidate of interest.

## Results

**Fig. 1:**
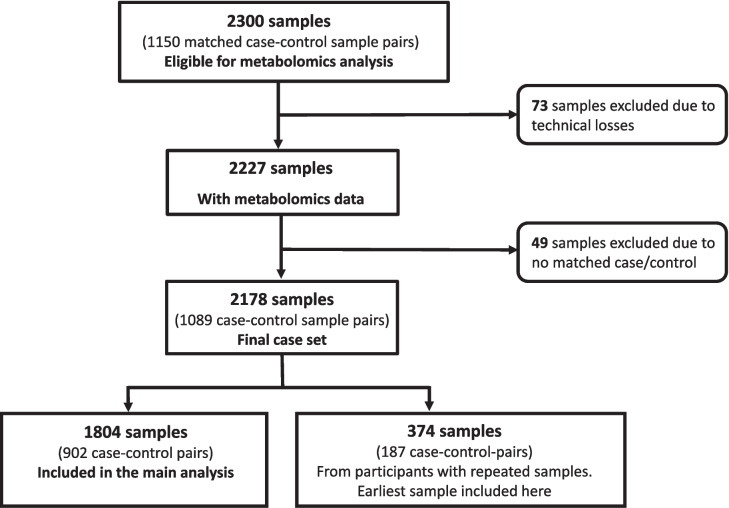
Flowchart of plasma samples eligible for LC–MS analysis. This nested case–control study included samples from two population-based cohorts within the Northern Sweden Health and Disease Study, which had recruited almost 142,000 participants as of end of May 2020. Participants who later developed colorectal cancers were identified through cancer registries, and control participants were selected with matching for sex, age at baseline, cohort, year of blood sampling and data collection, number of freeze–thaw cycles of the plasma samples, and fasting status at blood collection. After exclusion of samples due to technical losses, unavailable confounder data, and incomplete case–control pairs, 2178 samples (1089 case–control pairs) remained for statistical analysis. In total, 1804 samples were included in the main analysis after filtering out 374 earliest samples from participants with repeated sampling occasions

A total of 2300 plasma samples from 1150 case–control sample pairs were subject to LC–MS analysis. Some samples were excluded due to technical issues (low blood volume, instrument, or operation error), no available cohort data corresponding to the date of blood sampling, and lack of a case–control match due to exclusion for one of the above reasons. Of the remaining samples, 374 (from 187 case–control pairs with repeated sampling occasions) were excluded from the main statistical analysis since a more recent sample taken closer to diagnosis of the CRC case was available. The main analyses thus included 1804 samples from as many participants, of which 902 later developed CRC and 902 were matched controls (Fig. 1). The 374 earliest samples among the repeated measures (187 case–control pairs), which were excluded in the main analysis, were studied separately, to see if metabolite features associated with CRC in the main analysis showed the same pattern at time points further from CRC diagnosis. These samples were collected on average 9.7 years (standard deviation 2.5) prior to the later sample. For 744 CRC cases, tumor tissue was available and successfully analyzed for the *BRAF* V600E mutation, *KRAS* mutations, or MSI status.

### Descriptive statistics of key variables

The mean age at sample collection was 56.2 years$\pm 7.4$ (mean $\pm$ standard deviation), with an even sex distribution. The time from sample collection to diagnosis varied from 1 week to more than 26 years and on average cases were diagnosed with CRC 10.3 years after sample collection. Eighty-three percent of samples were collected after > 8 h of fasting, and 94% of samples had been thawed at most once prior to aliquoting for this study. The number of freeze and thaw cycles was higher in cases compared to controls (*p* < 0.001). Sixty-two percent of the participants were considered either overweight or obese (*BMI* > 25) among both cases and controls, and BMI was lower among the controls (*p* = 0.015). None of the other factors in Table 1 differed significantly between cases and controls at baseline. The distribution of CRC cases by site was 32% right-sided colon, 30% left-sided colon, and 38% rectum, and the distribution was approximately even between stages I–II and III–IV (Table 1). Of the CRC cases with molecular tumor data, 20% were *BRAF* mutated, 23% were *KRAS* mutated, and 13% were MSI. Observations that had missing values in an outcome variable (and their matched samples) were excluded from the respective subgroup analysis.

**Table 1:** Descriptive statistics of baseline variables in colorectal cancer cases and matched control samples

	Cases (*n* = 902)	Controls (*n* = 902)	*p*-value
Age at baseline	56.2 (7.4)	56.2 (7.4)	0.826
Age at diagnosis	66.5 (9.1)	-	-
Sex			1.000
Men	462 (51.2%)	462 (51.2%)	
Women	440 (48.8%)	440 (48.8%)	
BMI (kg/m^2^)^a^	26.7 (4.0)	26.3 (4.0)	0.015
Cohort			1.000
VIP	820 (91%)	820 (91%)	
MONICA	82 (9%)	82 (9%)	
Freeze and thaw cycles^b^			< 0.001
0	755 (84%)	752 (83%)	
1	79 (9%)	109 (12%)	
2	44 (5%)	40 (4%)	
3	24 (3%)	1 (0%)	
Fasting status			1.000
0–4 h	27 (3%)	27 (3%)	
4–8 h	125 (14%)	125 (14%)	
> 8 h	750 (83%)	750 (83%)	
Storage time (years)	20.9 (5.7)	20.9 (5.7)	0.976
Smoking status			0.221
Nonsmoker	364 (41%)	400 (45%)	
Former smoker	335 (38%)	307 (35%)	
Current smoker	187 (21%)	173 (20%)	
Missing	16	22	
Education			0.359
Elementary school	348 (39%)	369 (42%)	
Secondary school	397 (45%)	362 (41%)	
Postsecondary school	141 (16%)	157 (18%)	
Missing	16	14	
Diabetes			0.069
Yes	34 (4%)	20 (2%)	
No	863 (96%)	872 (98%)	
Missing	5	10	
Recreational physical activity			0.432
Never	373 (49%)	337 (44%)	
Now and then	201 (26%)	212 (28%)	
1–2 times/week	98 (14%)	103 (14%)	
2–3 times/week	61 (8%)	76 (10%)	
> 3 times/week	36 (5%)	30 (4%)	
Missing	133	144	
Alcohol intake (g/day)^c^	4.1 (5.0)	3.8 (4.7)	0.624
Tumor location			-
Proximal colon	286 (32%)	-	
Distal colon	266 (30%)	-	
Rectum	342 (38%)	-	
Missing	8	-	
Stage			-
Stages I–II	426 (51%)	-	
Stages III–IV	417 (49%)	-	
Missing	59	-	
*BRAF*^d^			-
Mutation	140 (20%)	-	
Wild type	565 (80%)	-	
Missing	197	-	
*KRAS*^d^			-
Mutation	154 23%)	-	
Wild type	504 (77%)	-	
Missing	244	-	
*KRAS/BRAF*^d^			-
*BRAF*	140 (22%)	-	
*KRAS*	154 (24%)	-	
Both wild type	354 (55%)	-	
Missing	254	-	
MSI status			-
MSS	568 (87%)	-	
MSI	87 (13%)	-	
Missing	247	-	

*BMI* body mass index. *VIP* Västerbotten Intervention Programme. *MONICA* Multinational Monitoring of Trends and Determinants in Cardiovascular Disease. *MSI* microsatellite instability. *MSS* microsatellite stable. Mean value and standard deviation are reported for continuous variable, whereas counts and percentage are presented for categorical variables. *P*-values were calculated using paired Wilcoxon signed-rank tests or chi-squared tests. ^a^Cases and controls had 5 and 7 missing values, respectively. ^b^Prior to aliquoting for shipment to lab. Of the case–control pairs, 92% were exactly matched on number of freeze–thaw cycles. ^c^Cases and controls had 98 and 101 missing values, respectively. ^d^Four of the missing values were samples with both *KRAS* and *BRAF* mutation, which were excluded in the subgroup analysis

### Plasma metabolomics for classification of CRC cases and controls

In the samples successfully analyzed by LC–MS, data were generated for 5015 metabolite features. To investigate the predictive potential of plasma metabolomics for CRC risk, we used a random forest classification approach. None of the potential confounders was selected in the variable selection step.

The predictive power was slightly higher in analyses limited to cases and matched controls with samples collected within 5 years prior to the case diagnosis, but the performance of the classification models remained low (Table 2). The results also showed poor separation between CRC cases and controls when men and women were analyzed separately. Since there were only minor differences in performance between the joint and sex-specific models (Additional Table 2), the sexes were combined for all downstream analyses. Sensitivity analysis regarding fasting time showed that exclusion of participants with < 8 h fasting also made negligible differences in prediction performance (Additional Table 2). Downstream analysis, therefore, included both fasting and non-fasting participants.

**Table 2:** Balanced error rates for random forest analysis of cases versus controls for samples collected at varying time intervals prior to diagnosis

	< 5 years	5–9 years	10–15 years	> 15 years	All samples
Balanced error rate^a,b^	0.43	0.49	0.50	0.54	0.46

Potential confounders (body mass index, smoking status, education level, diabetes, alcohol intake, and recreational physical activity) were included in the models, but none was selected in the built-in variable selection step. ^a^Balanced error rate for a two-class problem with expected BER by chance of 0.50. ^b^Since there is a 1:1 match between cases and controls (i.e., the data are perfectly balanced), the overall error rate is equal to the balanced error rate

Of all 5015 metabolite features, 480 were associated with CRC with a nominal *p* < 0.05 in the univariable analysis. After adjusting for multiple testing (*FDR* 0.25), seven features remained significant, all of which demonstrated inverse associations with CRC. Of these, five could not be annotated (levels 4–5) due to low feature intensity, resulting in absent or low quality MS2 fragmentation. One metabolite was tentatively annotated as pyroglutamic acid (level 2), and another feature was annotated as hydroxytigecycline (level 3). Inclusion of potential confounders in the conditional logistic regression models resulted in two significant metabolite features (Table 3), neither of which could be annotated. We also repeated the analyses for the seven features in the subset of 374 earliest samples (187 case–control pairs) from participants with repeated samples, from whom the sample collected closest to case diagnosis was included in the main analyses. In the univariable analysis, two features had nominal *p*-values < 0.05, one of which was hydroxytigecycline, and an additional two had *p*-values < 0.10. The directions of all associations were consistent with the main analyses, with lower concentrations in cases than controls. After inclusion of possible confounders in the models for the additional data set, two features had nominal *p*-values < 0.10, but none reached the significance level of 0.05.

**Table 3:** Metabolite features associated with CRC risk in conditional logistic regression-based analysis while controlling false discovery rate at 0.25

				Nominal *p*-value^a^ (FDR)				Odds ratio^b^ (95% confidence interval)			
Metabolite (ID level^c^; Identifier^d^)	LC–MS Mode	RT (s)	m/z Main fragment (additional fragments)	Univariable	Multivariable^e^	UV, earlier samples^f^	MV, earlier samples^g^	Univariable	Multivariable^f^	UV, earlier samples^f^	MV, earlier samples^g^
Unknown (5)	RP	40.79	191.0403 (169.0584)	0.0000 (0.0223)	0.0000 (0.0970)	0.1052	0.1500	0.7511 (0.6647–0.8488)	0.7633 (0.6743–0.8640)	0.8101 (0.6280–1.0451)	0.8235 (0.6321–1.0728)
Unknown phosphate C_3_H_9_N_2_O_5_P (4)	RP	40.61	207.0142	0.0000 (0.0792)	0.0001 (0.2458)	0.3782	0.3501	0.7420 (0.6447–0.8540)	0.7535 (0.6535–0.8688)	0.8718 (0.6426–1.1829)	0.8579 (0.6220–1.1832)
Unknown (5)	RP	407.11	696.2549 (697.2582)	0.0001 (0.1481)	0.0015 (0.4834)	0.0382	0.0710	0.7639 (0.6653–0.8772)	0.7930 (0.6871–0.9151)	0.7032 (0.5041–0.9810)	0.7254 (0.5119–1.0279)
Unknown (5)	RP	40.99	151.0478	0.0002 (0.1481)	0.0005 (0.4834)	0.0959	0.1724	0.8043 (0.7182–0.9007)	0.8150 (0.7262–0.9145)	0.7904 (0.5992–1.0425)	0.8207 (0.6178–1.0901)
Pyroglutamic acid (2; HMDB0000267)	RP	40.75	130.0500	0.0002 (0.1481)	0.0005 (0.4834)	0.0621	0.1104	0.7903 (0.6990–0.8936)	0.8025 (0.7087–0.9087)	0.7488 (0.5526–1.0148)	0.7751 (0.5669–1.0597)
Unknown (5)	RN	329.25	310.8388 (308.8412, 310.8388)	0.0002 (0.1481)	0.0007 (0.4834)	0.9240	0.8366	0.8107 (0.7257–0.9057)	0.8246 (0.7372–0.9223)	0.9883 (0.7753–1.2597)	1.0279 (0.7916–1.3347)
Hydroxytigecycline (3; ChEBI ID 142709)	RP	407.20	602.2791	0.0002 (0.1481)	0.0012 (0.4834)	0.0218	0.0504	0.7731 (0.6749–0.8857)	0.7961 (0.6932–0.9143)	0.6863 (0.4975–0.9466)	0.7139 (0.5094–1.0006)

*LC–MS* liquid chromatography-mass spectrometry. *RT* retention time. *FDR* false discovery rate. *UV* univariable. *MV* multivariable. *RP* reversed-phase positive. *RP* reversed-phase negative. ^a^*p*-values for likelihood ratio test. ^b^Odds ratios per one standard deviation increase in the exposure. ^c^Level of metabolite annotation according to the Schymanski scale [21]. ^d^Unique identifier at HMDB, ChEBI, or PubChem. ^e^Adjusted for potential confounders: body mass index, smoking status, education level, diabetes, alcohol intake, and recreational physical activity. ^f^A subset of 374 participants (187 case–control pairs) had repeated sampling occasions, of which the sample collected closest to case diagnosis was used in the main analyses. The analysis showed here used the earlier sample from these participants. ^g^Adjusted for potential confounders: body mass index, smoking status, education level, alcohol intake, and recreational physical activity (diabetes excluded from model due to only one sample with reported diabetes)

### Metabolomic profiles and subtypes of CRC

In addition to studying metabolites associated with overall CRC risk, we used the data to classify subjects according to clinical and molecular tumor subtypes. None of the potential confounders was selected in the variable selection step of the random forest models, with the exception of BMI, in the tumor location analysis restricted to samples taken < 5 years prior to diagnosis. In general, all models showed low predictive performance with balanced error rates (BER) close to what would be expected by chance, and no clear trend in performance was observed for CRC subtypes related to time to diagnosis (Table 4, Additional Table 3).

**Table 4:** Balanced error rate for classification using random forest model and stratified by follow-up time from sample collection to colorectal cancer diagnosis of cases

	< 5 years	5–9 years	10–15 years	> 15 years	All samples
**Three-level outcomes**					
Location (proximal, distal, rectal)^a^	0.72	0.63	0.66	0.59	0.62
*KRAS/BRAF* (*KRAS*, *BRAF*, both wt)^a^	0.68	0.67	0.70	0.64	0.67
**Two-level outcomes**					
Stage (stages I–II and stages III–IV)^b^	0.50	0.41	0.44	0.46	0.49
*KRAS* (mutation, wild type)^b^	0.50	0.47	0.42	0.44	0.50
*BRAF* (mutation, wild type)^b^	0.50	0.50	0.52	0.51	0.50
MSI (MSI, MSS)^b^	0.50	0.51	0.50	0.50	0.50

*wt* wild type. *MSI* microsatellite instability. *MSS* microsatellite stable. None of the potential confounders (body mass index, smoking status, education level, diabetes, alcohol intake, and recreational physical activity) was selected in the variable selection step of the random forest models, with the exception of body mass index in the tumor location analysis restricted to samples taken < 5 years prior to diagnosis. ^a^Balanced error rate for a three-class problem with expected BER by chance of 0.67. ^b^Balanced error rate for a two-class problem with expected BER by chance of 0.50

### Replication of previously reported CRC biomarker candidates

In 12 studies by 11 different research groups, blood, urine, or tumor tissue metabolomes were related to incident [8, 9, 25–28] or prevalent CRC [29–34], providing 297 potential metabolomic biomarker candidates (Additional Table 1). In our data, 37 features provided matches to exact mass (< 10 ppm) with 36 reported candidates and were associated with CRC risk (*p*-value in conditional logistic regression < 0.05).

Among these 37 features, six corresponded to the candidate of interest: L-tryptophan (level 2; *OR* (95% *CI*) 1.13 (1.00–1.28), *p* = 0.05), 3-hydroxybutyric acid (level 2; 1.14 (1.01–1.29), *p* = 0.04), sebacic acid (level 3; 0.85 (0.76–0.96), *p* = 0.01), 9,12,13-TriHOME (level 3; 1.24 (1.06–1.44), *p* = 0.01), valine (level 2; 1.20 (1.04–1.38), *p* = 0.01), and 13-OxoODE (level 3; 1.26 (1.04–1.51), *p* = 0.02). For the remaining features (*n* = 31), MS/MS fragmentation patterns were not matched in MassBank or well predicted by SIRIUS (*n* = 16) or obtained at all (*n* = 15) and could therefore not be confirmed.

## Discussion

Using an untargeted metabolomics approach to study potential plasma biomarkers of CRC risk in prospectively collected samples, we identified novel metabolite biomarkers associated with subsequent CRC risk and replicated some findings from previous studies. The metabolite profiles could not discriminate between individuals that later developed CRC and healthy control participants or between clinical and molecular tumor subtypes.

With respect to the novel findings, seven metabolite features were associated with CRC risk in the univariate analysis, two of which retained significance after adjusting for potential confounders. However, neither of these two features were successfully annotated, limiting further interpretation. In a separate, unadjusted, analysis using samples collected from 374 of the participants on average 9.7 years prior to the sample included in the main analysis, directions of associations were confirmed, and one annotated metabolite (hydroxytigecycline) was statistically significant at a nominal *p* < 0.05. The identified metabolites associated with CRC risk in our study might have relevance in terms of colorectal carcinogenesis. Pyroglutamic acid, also known as pidolic acid, is a derivative of L-glutamic acid and has been associated with dysregulation of glutamine and glutathione metabolism. Interestingly, higher levels of pyroglutamic acid have also been associated with use of the antibiotic flucloxacillin, through inhibition of 5-oxoprolinase in the glutamate/glutathione cycle [35]. Hydroxytigecycline, the other annotated feature, is also connected to antibiotic use, as it is a metabolite of the tetracycline family of broad-spectrum antibiotics. Several recent studies indicate that antibiotic use may increase the risk of colon cancer, particularly proximal colon cancer [36–38]. In our study, however, levels of pyroglutamic acid and hydroxytigecycline were lower in cases compared to controls, therefore not supporting the antibiotics hypothesis.

Although some studies identified alterations in metabolic pathways related to CRC [4], there are very few reports of validated metabolite biomarkers for early disease detection [5]. A limited number of studies have analyzed potential metabolite biomarkers for CRC in pre-diagnostic settings, most of which have focused on association to CRC risk by calculating odds ratios [8, 9, 39]. In a study based on an Asian population, a moderate discriminatory accuracy (*AUC* = 0.76) was obtained when classifying individuals that later developed CRC and their matched controls using conditional logistic regression (8). However, the results were not evaluated using a test set or cross validation, and over-fitting was noted as a potential issue. Another study based on 254 incident CRC cases and 254 matched controls found suggestive associations between some metabolites and CRC risk, though none reached significance after adjusting for multiple testing. Also, similar to our findings, random forest analysis resulted in poor predictive power of future cases (error rate = 0.497) [39]. Taken together, the results of previous studies and the current investigation suggest that the circulating metabolome is likely to be a poor predictor of future CRC.

In addition to the main, agnostic approach in our study, we also attempted to replicate previously reported candidate metabolite markers of CRC or CRC risk. Of 37 markers with exact mass matches in our data, we found associations with CRC for six metabolites of which two were of special interest: L-tryptophan and 3-hydroxybuyric acid. Tryptophan (L-tryptophan) is an essential amino acid, and lower levels of free tryptophan in plasma have been linked to progression in several cancer types including CRC [40]. Tryptophan metabolism plays an important role in the regulation of the immune system as catabolism of tryptophan through the kynurenine pathway inhibits T-cell proliferation [41]. Two previous studies reported inverse associations between L-tryptophan blood levels and CRC [40, 42]. In contrast, we observed a positive association, i.e., individuals who later developed CRC had higher levels of L-tryptophan in plasma compared to controls. The second metabolite of interest was 3-hydroxybutyric acid (beta-hydroxybutyric acid), levels of which were higher in our prospective CRC cases compared to the controls. In an earlier study of 3-hydroxybutyric acid, using urine samples from a Canadian cohort, higher levels were also observed in CRC cases compared to controls, but the results could not be replicated in American patients [28]. Interestingly, 2-hydroxy-3-methylbutyric acid, which is a derivative of 3-hydroxybutyric acid, was recently identified as a biomarker of habitual alcohol intake and associated with risk of hepatocellular carcinoma (HCC) and pancreatic cancer in two large independent cohorts (EPIC and ATBC) [43]. This finding is well in line with our results as high alcohol consumption is also a known risk factor for CRC.

A main limitation of metabolomics research using biobanked samples is the sensitivity of the methodology to pre-analytical sample management. Factors including freeze–thaw cycles and storage time and temperature are known to affect the measurable metabolome and might distort the results [44, 45]. In our study, the samples were collected and handled according to strict protocols and were matched between cases and controls with respect to relevant pre-analytical factors, such as fasting status (only 3% of the participants had fasted < 4 h, 83% fasted > 8 h), thus reducing bias. We did not conduct analyses stratified by pre-analytical factors because of limited power for such subgroups and to reduce the risk of chance findings due to multiple testing. However, the sensitivity analysis limiting the random forest analysis to participants who had fasted > 8 h did not affect predictive power. Overall, the sample size in our study should be large enough to discover pre-diagnostic disease markers even with potential noise caused by variability in pre-analytical factors. Other possible limitations of the study are the use of self-reported data for some of the lifestyle-related potential confounders, minor changes in the questionnaire over time, and the lack of information regarding some CRC risk factors, such as family history and use of nonsteroidal anti-inflammatory drugs (NSAIDs). That said, the confounder data, including the metabolic factors BMI (measured by a health professional) and diabetes, were strengths of the study. This study is one of the largest CRC metabolomics studies to date, and in addition to including a large number of well-characterized CRC cases and matched controls from population-based cohorts, the main strengths are the prospective design, with samples collected at varying time points prior to case diagnosis, and the clinical and molecular tumor data for the CRC cases. The sampling protocol in VIP and MONICA was designed with needle-to-freezer time < 60 min, including mainly fasting samples and storage time of < 1 week at − 20 °C prior to long-term storage at − 80 °C, thus ensuring uniform and high sample quality well suited for metabolomics analysis. Earlier reports agree that storage of samples up to 1 week in − 20 °C freezers have negligible effects on the plasma metabolome [46, 47]. We were able to take into consideration baseline assessments of several CRC risk factors. Finally, we applied a rigorous procedure for obtaining high-quality untargeted LC–MS metabolomics data. A limitation of the untargeted approach was the low intensity of several of the reported features, with limited MS/MS fragmentation data, consequently limiting the possibilities for accurate identification. However, reporting of unannotated features is important for availability in future research.

## Conclusions

In conclusion, the findings of this large population-based, nested case–control study suggest that although circulating metabolites may provide etiologic insight for further investigation, they demonstrate the importance of validation studies and suggest that the circulating metabolome alone probably cannot achieve a discriminatory performance sufficient for clinical application in risk stratification or precision screening for CRC.

### Supplementary Information

**Additional file 1:** **Additional notes.** Parameters used for the R packages XSMS, RAMClustR and the software Sirius+CSI:FingerID.

**Additional file 2: Additional Table 1.** Metabolites previously reported to be associated with CRC.

**Additional file 3: Additional Table 2.** Sensitivity analysis regarding sex and fasting status. Overall error rate (OER) for random forest models stratified by sex and for models restricted to samples with at least 8h fasting . Since the groups are perfectly balanced, the balanced error rate is the same as the OER. None of the variables body mass index, smoking, education level, diabetes, alcohol intake and recreational physical activity were selected by the built-in variable selection.

**Additional file 4: Additional Table 3.** Overall error rate stratified by follow-up time. Overall error rate. Observed performance (in bold) and performance expected by chance for colorectal cancer subtype analysis using random forest models stratified by follow-up time from sample collection to colorectal cancer diagnosis of cases. Potential confounders (body mass index, smoking, education level, diabetes, alcohol intake and recreational physical activity) and the matching variables (cohort, baseline age, gender, freeze/ thaw cycles, year of blood sampling and fasting status) were added in the model, but none of the variables were selected in the random forest models except for body mass index in the analysis of tumor location in the group <5 years.

## Data Availability

Due to legal restrictions imposed by the Swedish Data Protection Authority, we are not allowed to share individual-level data freely. Instead, any researcher interested in the individual-level data may apply for access at the Biobank Research Unit at Umeå University (https://www.umu.se/en/biobank-research-unit/provsamlingar-och-register/northern-sweden-health-and-disease-study-vip-monica-and-the-mammography-screening-project/). The application will be subject to ethical review and assessment by an expert committee. R-codes used for analyzing the data are available at https://github.com/LindaVi/CRC_Metabolomics.

## Abbreviations

CRC: Colorectal cancer
NSHDS: Northern Sweden Health and Disease Study
LC–MS: Liquid chromatography–mass spectrometry
VIP: Västerbotten Intervention Programme
MONICA: Multinational Monitoring of Trends and Determinants in Cardiovascular Disease
MSI: Microsatellite instable
MSS: Microsatellite stable
sQCs: Study-specific quality control samples
RP: Reversed-phase positive
RN: Reversed-phase negative
CV: Coefficient of variation
CL: Confidence level
FDR: False discovery rate
OR: Odds ratios
CI: Confidence interval
BMI: Body mass index
OER: Overall error rate
BER: Balanced error rate
NSAIDs: Nonsteroidal anti-inflammatory drugs
RT: Retention time
UV: Univariable
MV: Multivariable
wt: Wild type
