# Supplementary material for: Untargeted plasma metabolomics and risk of colorectal cancer—an analysis nested within a large-scale prospective cohort
Source: Cancer Metab. 2023 Oct 17;11:17. doi: 10.1186/s40170-023-00319-x (PMC10583301; doi:10.1186/s40170-023-00319-x)
Supplement: Supplementary file 1 — Additional file 1: Additional notes. Parameters used for the R packages XSMS, RAMClustR and the software Sirius+CSI:FingerID. [file 40170_2023_319_MOESM1_ESM.docx]

| **Parameters used for XCMS^a^** |
| --- |
| - Reverse phase Negative (RN): CentWaveParam(peakwidth = c(7, 60), noise=500, ppm=22, mzdiff=0.0014, prefilter=c(3, 5000), integrate=1); PeakGroupsParam(minfrac=0.95, smooth="loess", span=0.4, family="gaussian"); PeakDensityParam(minfrac=0.4, bw=1.5, binSize=0.015). |
| - Reverse phase Positive (RP): CentWaveParam(peakwidth=c(7.5, 62), noise=500, ppm=20, mzdiff=-0.003, prefilter=c(3, 5000), integrate=1); PeakGroupsParam(minfrac=0.95, smooth="loess", span=0.4, family="gaussian"); PeakDensityParam(minfrac=0.4, bw=1.5, binSize=0.015). |
| **Parameters used for RAMClustR** |
| - The parameters were manually optimized as follows: st=0.5, sr=0.35, which resulted in 775 and 820 clusters for RN and RP modes, respectively. For each cluster, feature with the highest intensity that was considered to be the representative of this cluster and together with 1616 and 1824 singletons (i.e. features not included in any cluster) for the two modes were subsequently used in multivariate modelling.   **Parameters used for Sirius+CSI:FingerID v4.8.2**   - SIRIUS + ZODIAC + CSI:FingerID used for all predictions. All set to default settings except for “Consider only formulas in DBs:” option in the Sirius settings which was set to “Bio Database”.   **Parameters used for MetFrag**   - Database settings: Pubchem Lite, Search ppm = 5 - Candidate Filter & Score Settings: Exact spectral similarity (MoNA) - Fragmentation Settings & Processing: Default settings   **Parameters used for HMDB**   - DPPM = 10, Adducts = M+H, M-H   **Parameters used for in-house library**   - Settings: DPPM = 5, dRT = 15s, mzWeight = 0, intWeight = 1 - Cut-offs: Cosine sim. score = 0.9, n matching peaks: 2 |
| ^a^ Other parameters in XCMS were default values. |
