# Supplementary material for: Untargeted plasma metabolomics and risk of colorectal cancer—an analysis nested within a large-scale prospective cohort
Source: Cancer Metab. 2023 Oct 17;11:17. doi: 10.1186/s40170-023-00319-x (PMC10583301; doi:10.1186/s40170-023-00319-x)
Supplement: Supplementary file 3 — Additional file 3: Additional Table 2. Sensitivity analysis regarding sex and fasting status. Overall error rate (OER) for random forest models stratified by sex and for models restricted to samples with at least 8h fasting . Since the groups are perfectly balanced, the balanced error rate is the same as the OER. None of the variables body mass index, smoking, education level, diabetes, alcohol intake and recreational physical activity were selected by the built-in variable selection. [file 40170_2023_319_MOESM3_ESM.docx]

| OER | <5 years | 5-9 years | 10-15 years | >15 years | All samples |
| --- | --- | --- | --- | --- | --- |
| Men | 0.42 | 0.43 | 0.41 | 0.56 | 0.45 |
| Women | 0.40 | 0.52 | 0.58 | 0.44 | 0.52 |
| Excluded fasting <8h | 0.43 | 0.52 | 0.48 | 0.63 | 0.46 |
