# Supplementary material for: Untargeted plasma metabolomics and risk of colorectal cancer—an analysis nested within a large-scale prospective cohort
Source: Cancer Metab. 2023 Oct 17;11:17. doi: 10.1186/s40170-023-00319-x (PMC10583301; doi:10.1186/s40170-023-00319-x)
Supplement: Supplementary file 4 — Additional file 4: Additional Table 3. Overall error rate stratified by follow-up time. Overall error rate. Observed performance (in bold) and performance expected by chance for colorectal cancer subtype analysis using random forest models stratified by follow-up time from sample collection to colorectal cancer diagnosis of cases. Potential confounders (body mass index, smoking, education level, diabetes, alcohol intake and recreational physical activity) and the matching variables (cohort, baseline age, gender, freeze/ thaw cycles, year of blood sampling and fasting status) were added in the model, but none of the variables were selected in the random forest models except for body mass index in the analysis of tumor location in the group <5 years. [file 40170_2023_319_MOESM4_ESM.docx]

|  | Location  Exp. Obs. | | Stage  Exp. Obs. | | *KRAS*  Exp. Obs. | | *BRAF*  Exp. Obs. | | | *KRAS*/*BRAF*  Exp. Obs. | | MSI  Exp. Obs. | |
| --- | --- | --- | --- | --- | --- | --- | --- | --- | --- | --- | --- | --- | --- |
| <5 years | 0.61 | **0.67** | 0.50 | **0.50** | 0.30 | **0.31** | | 0.09 | **0.09** | 0.40 | **0.42** | 0.08 | **0.08** |
| 5-9 years | 0.61 | **0.58** | 0.49 | **0.41** | 0.25 | **0.23** | | 0.20 | **0.20** | 0.44 | **0.44** | 0.14 | **0.14** |
| 10-15 years | 0.66 | **0.65** | 0.50 | **0.44** | 0.22 | **0.17** | | 0.28 | **0.30** | 0.53 | **0.57** | 0.13 | **0.13** |
| >15 years | 0.61 | **0.53** | 0.49 | **0.45** | 0.29 | **0.24** | | 0.27 | **0.28** | 0.57 | **0.55** | 0.20 | **0.19** |
| All samples | 0.64 | **0.59** | 0.50 | **0.49** | 0.24 | **0.24** | | 0.20 | **0.21** | 0.47 | **0.47** | 0.14 | **0.14** |

MSI – Microsatellite instability

Exp – Expected

Obs - Observed
